# MycoResistance: a curated resource of drug resistance molecules in *Mycobacteria*

**DOI:** 10.1093/database/baz074

**Published:** 2019-07-10

**Authors:** Enyu Dai, Hao Zhang, Xu Zhou, Qian Song, Di Li, Lei Luo, Xinyu Xu, Wei Jiang, Hong Ling

**Affiliations:** 1 Department of Microbiology, Harbin Medical University, Xuefu Road, Nangang District, Harbin, China; 2 Wu Lien-Teh Institute, Harbin Medical University, Xuefu Road, Nangang District, Harbin, China; 3 Department of Parasitology, Harbin Medical University, Xuefu Road, Nangang District, Harbin, China; 4 Heilongjiang Provincial Key Laboratory of Infection and Immunity, Xuefu Road, Nangang District, Harbin, China; 5 Key Laboratory of Pathogen Biology, 194 Xuefu Road, Nangang District, Harbin, P. R. China; 6 Department of Biomedical Engineering, College of Automation Engineering, Nanjing University of Aeronautics and Astronautics, Jiangjun Avenue, Jiangning District, Nanjing, P.R. China

## Abstract

The emergence and spread of drug-resistant *Mycobacterium tuberculosis* is of global concern. To improve the understanding of drug resistance in *Mycobacteria*, numerous studies have been performed to discover diagnostic markers and genetic determinants associated with resistance to anti-tuberculosis drug. However, the related information is scattered in a massive body of literature, which is inconvenient for researchers to investigate the molecular mechanism of drug resistance. Therefore, we manually collected 1707 curated associations between 73 compounds and 132 molecules (including coding genes and non-coding RNAs) in 6 mycobacterial species from 465 studies. The experimental details of molecular epidemiology and mechanism exploration research were also summarized and recorded in our work. In addition, multidrug resistance and extensively drug resistance molecules were also extracted to interpret the molecular mechanisms that are responsible for cross resistance among anti-tuberculosis drugs. Finally, we constructed an omnibus repository named MycoResistance, a user friendly interface to conveniently browse, search and download all related entries. We hope that this elaborate database will serve as a beneficial resource for mechanism explanations, precise diagnosis and effective treatment of drug-resistant mycobacterial strains.

## Introduction

Although the infection rate of *Mycobacterium tuberculosis* has declined globally, the threat of tuberculosis to public health has worsened with the emergence of drug resistance in *M. tuberculosis* strains, particularly multidrug-resistant (MDR) and extensively drug-resistant (XDR) strains (1, 2). Resistance to rifampicin and isoniazid is classified as MDR tuberculosis, and additional resistance to the fluoroquinolones and any of the injectable drugs (amikacin, kanamycin or capreomycin) used to treat MDR strains is termed XDR tuberculosis. Failure to identify patients with drug-resistant tuberculosis can lead to increased mortality, nosocomial outbreaks and drug resistance expansion (3). Meanwhile, the second-line drugs that are commonly used to treat these resistant cases are more expensive and toxic, and the sophisticated infrastructure for drug susceptibility detection is not readily available in resource-limited settings (4). Additionally, inadequate treatment risks amplification of resistance to further drugs and may expand opportunities for transmission (5).

The primary vehicle driving drug resistance in *M. tuberculosis* is the acquisition of mutations in genes that code for drug targets or drug-activating enzymes (1), such as drug resistance-associated mutations in rpoB for rifampicin (6), in katG and mabA-inhA operons for isoniazid (7, 8), in embB for ethambutol (9), in pncA for pyrazinamide (10), etc. Moreover, changes in efflux pump regulation may have an impact on the emergence of resistance (11), and putative compensatory mechanisms to overcome fitness impairment coincidental with the acquisition of resistance have been described for some drugs (12). In a separate mode, the expression dysregulation of some mycobacterial genes was reported to play significant roles in the drug susceptibility of *Mycobacteria*. For instance, the expression levels of ddrA and ddrB were upregulated in XDR *M. tuberculosis* strains compared to drug-susceptible strains (13), and overexpression of ddrA and ddrB in *Mycobacterium smegmatis* conferred the resistance of the isolates to multiple clinically relevant, structurally unrelated antibiotics (14). In summary, it is paramount to explore the molecular mechanisms of drug resistance in *Mycobacteria*, in order to limit the spread of drug-resistant strains, reduce treatment duration, minimize adverse effects and improve treatment outcomes.

Rapid and effective methods for the diagnosis of drug-resistant tuberculosis are vital so that clinicians can make appropriate decisions regarding drugs that will be most effective for treatment. At present, drug susceptibility testing (DST) for *M. tuberculosis* can be performed using conventional phenotypic methods or molecular detection of genetic determinants related to drug resistance (15, 16). As a gold standard approach, phenotypic DST methods in solid medium and liquid medium are easily reproducible, and their results correlate well with clinical courses. However, these conventional assays are time-consuming and challenged by technical difficulties and biosafety issues (17, 18). Meanwhile, the development of molecular technologies has caused the emergence of genotypic tools for the testing of drug-resistant tuberculosis, ranging from commercial systems to whole-genome sequencing approaches. Currently, several molecular kits are available to assess *M. tuberculosis* resistance based on line probe assays or real-time polymerase chain reaction, which allow for the prediction of drug resistance in clinical specimens within one working day (15, 18, 19).

In brief, a database that stores the molecules and their potential roles in drug susceptibility is valuable for the prevention and treatment of tuberculosis. However, the related information is scattered in a massive body of literature, which is not conducive to study deeply and transfer the discoveries into clinical practice. Accordingly, we constructed an omnibus repository named MycoResistance, furnishing a user-friendly interface for convenient retrieval of drug resistance molecules (including coding genes and non-coding RNAs) in *Mycobacteria*. In addition, more experimental details of molecular epidemiology and mechanism exploration research were extracted and summarized in our resource. In particular, the MycoResistance database also retained MDR- and XDR-associated molecules to improve the understanding of regulatory roles that are responsible for cross resistance among anti-tuberculosis drugs. Our elaborate database specially designed to indicate relationships between drug resistance and molecules could serve as a significant resource for research on the functional mechanisms and precise treatment of drug-resistant mycobacterial strains.

## Materials and Methods

### Study selection

A literature search was conducted of all publications evaluating associations between drug resistance and molecules in *Mycobacteria*. In total, 32 keywords were used to search the PubMed database (Supplementary Figure 1). The manual curation was restricted to studies that were published from 1959 to October 1, 2018, including those studies available online prior to publication. Research works were selected if they met the following predetermined criteria: (i) published in English, (ii) presented original data and (iii) assessed drug resistance based on phenotypic and genotypic DST methods. Both molecular epidemiology and mechanism exploration research were reviewed in this work.

### Information extraction

To clearly display the associations between drug resistance and molecules in *Mycobacteria*, our database extracted corresponding details for molecular epidemiology and mechanism exploration research. For molecular epidemiology research, three aspects of information, including the literature summary, epidemiology summary and molecular change details, were collected from each study. In the epidemiology summary, we gathered epidemiological characteristics of tuberculosis patients (tuberculosis type, geographical origin, drug resistance pattern and lineage distribution), as well as the phenotypic and molecular detection methods used. Simultaneously, complete descriptions of drug resistance-associated mutations (position of mutation, nucleotide or amino acid changes, type of mutation and mutation frequency in resistant and susceptible isolates) were obtained in the database.

Both the mechanism summary and literature summary were retrieved and summarized to characterize the drug resistance molecule relationships from mechanism exploration research. The mechanism summary consists of four sections: the research strategy used (such as gene knock-in method or case-control method), the experiment model (*in vitro* or *in vivo* models), the detection methods (including phenotypic and genotypic drug susceptibility tests) and the direct evidence.

In addition, three kinds of external website links, including PubMed, PubChem and Mycobrowser, were built in MycoResistance. For the PubMed website, we obtained the PubMed ID for each entry and appended it to the end of the link https://www.ncbi.nlm.nih.gov/pubmed/ to finish the PubMed link construction. Moreover, the basic descriptions (formula, weight, grade, route and PubChem CID) of each compound were achieved from the PubChem database, and PubChem external links were constructed by adding the PubChem CID to the end of the URL https://pubchem.ncbi.nlm.nih.gov/compound/. Similarly, we gained the genomic location, gene length, protein length and function category for each molecule from the Mycobrowser (https://mycobrowser.epfl.ch/) and provided the corresponding links to the website.

### Webserver construction

The current version of MycoResistance was established based on three major software components: an Apache Tomcat web server, a MySQL relational database and Java-based computational services. All data in MycoResistance were stored and managed using MySQL (version 5.5.58). Additionally, the web interfaces were built in Java Sever Pages, the data-processing programs were written in Java (version 1.6.0) and the web services were constructed using Apache Tomcat.

## Results

### Data curation and database content

**Figure 1:**
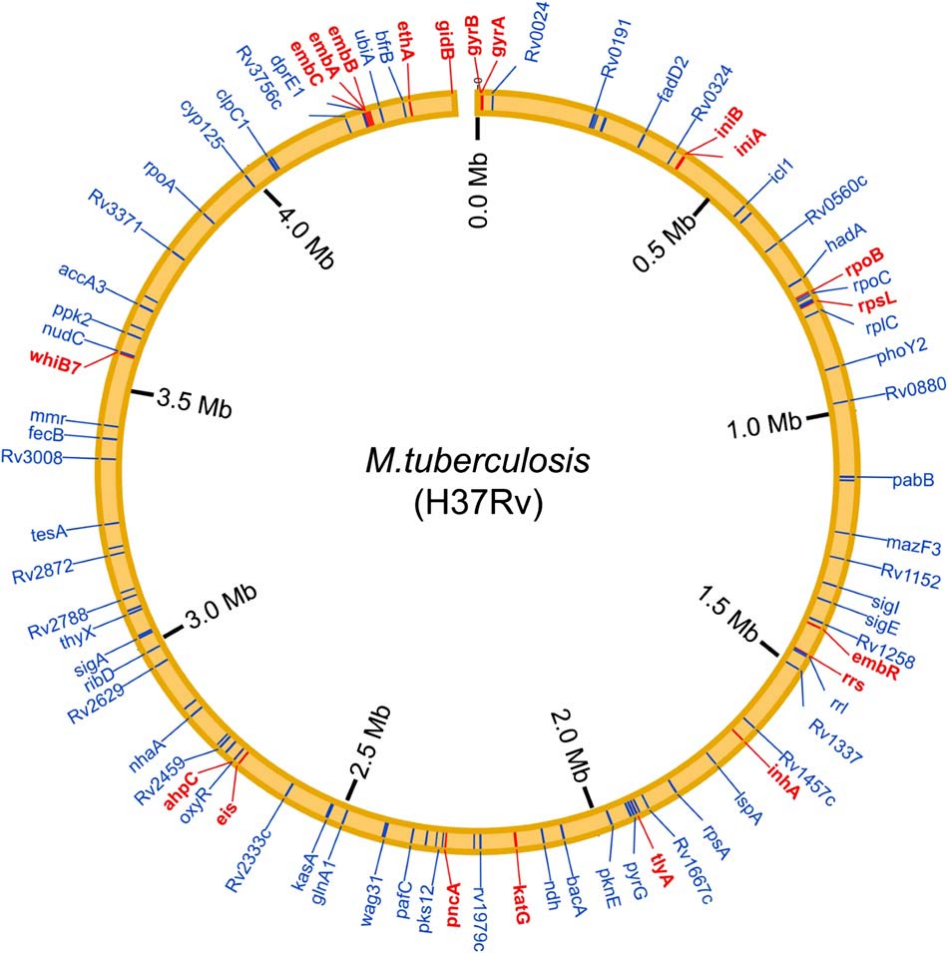
Genomic distribution of the curated drug resistance-associated molecules in *M. tuberculosis*. Drug resistance-associated molecules that were detected in the molecular epidemiology research are shown by red lines, whereas that were identified in the mechanism exploration research are shown by blue lines.

To systematically obtain high-quality information about drug resistance-associated molecules in *Mycobacteria*, we searched the PubMed database with a list of keywords (Supplementary Figure S1), and reviewed 3705 published articles in detail. A total of 1707 curated associations between 73 compounds and 132 molecules in 6 mycobacterial species (including *M. tuberculosis*, *M. smegmatis*, *Mycobacterium bovis*, *Mycobacterium marinum*, *Mycobacterium leprae* and *Mycobacterium abscessus*) were manually collected from 465 studies (Table 1). Of the 132 molecules, there are 127 coding genes and 5 non-coding RNAs. Moreover, promoters (non-coding region) of eight coding genes were reported to be associated with anti-tuberculosis drug resistance. Especially, the genomic distribution of curated drug resistance molecules in *M. tuberculosis* is shown in the chromosome-based circos plot (Figure 1). Many well-known drug resistance-associated molecules (such as rpoB, katG, embB, gyrA, pncA and rrs) were detected by the molecule epidemiology research, and multiple novel markers were discovered in the mechanism exploration research. Correspondingly, all acquired records are divided into two parts: ‘Molecular Epidemiology’ and ‘Mechanism Exploration’ in this study. We also gathered comprehensive descriptions of epidemiology and changes in molecules for MDR and XDR isolates. Moreover, we obtained and presented additional annotations of compounds and molecules (such as basic information about the compounds, genomic annotation of molecules and so on) and external links to other databases, including articles in the PubMed database, compounds in the PubChem and DrugBank database (20) and mycobacterial molecules in the Mycobrowser database (21).

**Table 1:** Summary of curated records that were stored in MycoResistance database

**Species**	**No. of record**	**No. of compound**	**No. of molecule**	**No. of literature**
*M. tuberculosis*	1596	59	99	434
*M. smegmatis*	65	24	38	27
*M. bovis*	27	16	13	13
*M. marinum*	7	5	4	2
*M. leprae*	5	5	1	1
*M. abscessus*	7	7	1	1

The ‘Molecular Epidemiology’ records are mainly focused on the research that characterized the diversity of drug resistance molecules and the frequency of mutations in resistant and susceptible *Mycobacterium* strains. In this section, MycoResistance not only displays detailed genomic variation data (mutation position, nucleotide or amino acid change, mutation type and frequency) for clinical isolates with phenotypic resistance profiles but also shows the epidemiological characteristics of these clinical isolates, containing the tuberculosis type, geographic origin, drug resistance pattern and *M. tuberculosis* genotype (lineages) distribution. Similarly, an epidemiological summary and molecular change details of MDR and XDR strains are presented in the ‘Molecular Epidemiology’ section.

**Figure 2:**
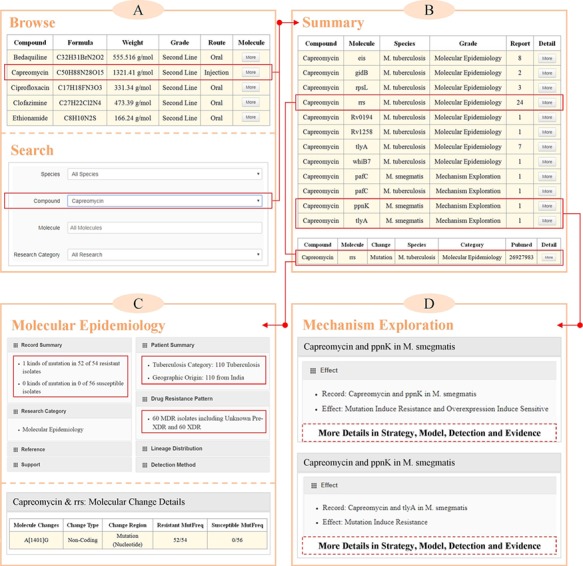
Case study of querying molecules that were associated with the resistance to capreomycin in MycoResistance. (A) Queries on capreomycin resistance-associated molecules based on the ‘Browse’ or ‘Search’ pages. (B) A complete list of curated associations between capreomycin and mycobacterial molecules on the ‘Summary’ page. (C) Example of capreomycin resistance molecule, rrs, in the ‘Molecular Epidemiology’ section of ‘Result’ page. (D) Examples of capreomycin resistance molecules, ppnK and tlyA, in the ‘Mechanism Exploration’ section of ‘Result’ page.

The studies that uncovered the potential roles of the mycobacterial molecules in anti-tuberculosis drug resistance are compiled in the ‘Mechanism Exploration’ section. With direct evidence, each record of ‘Mechanism Exploration’ summarizes the effect of a molecule on the drug resistance, such as ‘mutation induces resistance’ or ‘deletion induces susceptibility’. In addition, several key details of validated experiments (research strategy, experimental model and drug-susceptibility testing method) are shown in the MycoResistance database.

### User interface

MycoResistance furnishes a user-friendly web interface that enables users to browse, search and retrieve the records of drug resistance molecule associations in *Mycobacteria*. In our database, there are four main pages: ‘Browse’, ‘Search’, ‘Download’ and ‘Result’ (Supplementary Figure S2). Additionally, our database offers a detailed operation tutorial on the ‘Manual’ page to help new users make better use of the platform.

On the ‘Browse’ page, users can scan all compounds in the database by clicking a specified compound name and then review a full report of curated molecules that are associated with drug resistance. Four kinds of query option (‘Species’, ‘Compound’, ‘Molecule’ and ‘Research Category’) are provided on the ‘Search’ page. Users can look for information based on separate query or composite query in MycoResistance. Our resource also offers direct links to view the records of molecular epidemiology studies for MDR and XDR strains. Finally, a local copy of this database and other annotation files can be achieved on the ‘Download’ page.

After selecting a compound on the ‘Browse’ or ‘Search’ pages, a complete list of drug resistance molecules across different species is returned. By clicking the ‘More’ button, users can get access to full details of ‘Molecular Epidemiology’ and ‘Mechanism Exploration’ records on the ‘Result’ page. For the molecular epidemiology research, MycoResistance exhibits each entry in three parts, including ‘Literature Summary’, ‘Epidemiology Summary’ and ‘Molecular Change Details’ sections. Similarly, both ‘Mechanism Summary’ and ‘Literature Summary’ are provided to elaborate the regulatory mechanism of the molecule underlying the drug resistance. Additionally, the essential information of the compound and molecule are shown on the right side of the ‘Result’ page.

### Case study

As a commonly used second-line injectable agent, the cyclic polypeptide capreomycin is currently applied to the treatment of drug-resistant tuberculosis. Through binding to the bacterial ribosome and then modifying the structure of 16S rRNA, capreomycin can inhibit the protein synthesis of *Mycobacteria*. To comprehensively investigate molecular determinants of capreomycin resistance, users can query this drug based on the ‘Browse’ or ‘Search’ pages in the MycoResistance database (Figure 2A). As illustrated in Figure 2B, there are 12 records from molecular epidemiology and mechanism exploration research, which cover 10 mycobacterial molecules in *M. tuberculosis* and *M. smegmatis*. By clicking the ‘More’ button, users can access additional details for each entry.

More specifically, a total of eight molecules (rrs, eis, gidB, rpsL, tlyA, whiB7, Rv0194 and Rv1258) were discovered to be related to resistance to capreomycin in the ‘Molecular Epidemiology’ section of MycoResistance. Taking rrs as an example, there are 24 published works that have revealed the mutations of rrs in the capreomycin-resistant and capreomycin-susceptible isolates (Figure 2B). Among these studies, Kambli *et al*. (22) gathered 110 *M. tuberculosis* clinical isolates (include 60 XDR strains) from India and performed phenotypic and genotypic DST for each strain based on MGIT 960 system, GenoType MTBDRsl assay and DNA sequencing method (Figure 2C). The A–G polymorphism at position 1401 of the rrs was detected in the 52 of 54 capreomycin-resistant strains, but not in the susceptible strains (22).

As another example, the roles of pafC, ppnK and tlyA in resistance to capreomycin were elaborated in the ‘Mechanism Research’ section of the MycoResistance database (Figure 2B). For instance, as shown in Figure 2D, Du *et al.* (23) constructed an *M. smegmatis* transposon mutant library and isolated a mutant (named C4) with 4-fold increased capreomycin resistance. Based on the whole genome sequencing method, a mutation (A to G substitution) in the overlap region between ppnK and tlyA was detected in the genome of C4. Similarly, overexpression of ppnK in both *Escherichia coli* and *M. smegmatis* conferred subtle susceptibility to capreomycin (23). Using these tools, researchers in related fields can make use of our database to fully comprehend the molecular level and regulatory process of key molecules in mycobacterial strains.

## Discussion

In recent years, researchers have been trying to decipher the precise molecular mechanisms involved in antibiotic resistance. Consequently, studies focused on drug resistance-associated molecules in *Mycobacteria* have accumulated rapidly in the past decade. However, most associations between drug resistance and molecules were dispersed in many independent research works. It becomes increasingly obvious that an integrated collection of drug resistance molecule relationships is crucial for clear understanding of the regulatory mechanisms underlying anti-tuberculosis drug resistance. Thus, we developed MycoResistance, a user-friendly database that provides a comprehensive resource regarding anti-tuberculosis drug resistance molecules in several mycobacterial species with detailed annotations.

To date, several databases have been constructed to uncover the associations between drugs and molecules in *M. tuberculosis*. For instance, PolyTB (24) and tbvar (25) systemically identified genomic variations based on large-scale whole genome sequencing data of *M. tuberculosis* isolates, and then annotated the drug resistant variants by using TBDreamDB (26), which is a free online resource that gathered information on the anti-tuberculosis drug resistance-associated mutations. Although very helpful, the TBDReaM database has not been updated since April 2010, and its usage remains time-consuming because it demands prior processing of the nucleotide sequences to detect mutations and the database cannot be easily interrogated due to its relaxed grammatical conception. In addition, TuberQ (27) and TDR Targets (28) were designed and developed to facilitate the identification and prioritization of molecular targets for drug development in *M. tuberculosis*. Compared with these databases, MycoResistance applies a manual curation strategy to collect reliable relationships between molecules and the resistance to compounds in *Mycobacteria*. Both genome variants and expression changes that play significant roles in drug resistance were retrieved and represented in the database, which elaborated comprehensive mechanisms underlying the anti-tuberculosis drug resistance.

As stated above, the drug resistance molecule relationships in the current release were collected through searching the PubMed database with a list of keywords. Although we have gathered 1707 curated associations from 465 literature citations, such a mechanism suffers from the lack of systematization and comprehensiveness. Therefore, we plan to update our database regularly in the future. With constant expansion and optimization, MycoResistance will become a more comprehensive and convenient resource regarding associations between anti-tuberculosis drug resistance and mycobacterial molecules from molecular epidemiology and mechanism exploration studies.

In summary, recent research has shed light on the molecular mechanisms involved in resistance to anti-tuberculosis drugs. Through collecting and interpreting these studies, MycoResistance not only provides a significant reference for molecular biologists to understand and explain the regulatory mechanisms of drug susceptibility in *Mycobacteria*, but also offers potential biomarkers for controlling, diagnosing and treating drug-resistant tuberculosis. In the future, we will continue to manually mine newly validated drug resistance molecule associations. Meanwhile, we plan to integrate more functional and regulatory annotations for each record and develop additional tools for predicting the susceptibility of clinical strains to anti-tuberculosis drugs. MycoResistance will become a valuable resource for mechanism explorations, precise diagnosis and effective treatment of drug-resistant mycobacterial strains.

## Supplementary Material

Supplementary_File_baz074

## Notes

**Database URL:** http://www.hmulinglab.org/MycoResistance/
