# Supplementary material for: MycoResistance: a curated resource of drug resistance molecules in Mycobacteria
Source: Database (Oxford). 2019 Jul 10;2019:baz074. doi: 10.1093/database/baz074 (PMC6619405; doi:10.1093/database/baz074)

MycoResistance: A Curated Resource of Drug Resistance Molecules in *Mycobacteria*

Enyu Dai[1], Hao Zhang[1], Xu Zhou[2], Qian Song[2], Di Li[1], Lei Luo[1], Xinyu Xu[1], Wei Jiang[2,*] and Hong Ling[1,*]

[1] Department of Microbiology; Wu Lien-Teh Institute; Department of Parasitology, Harbin Medical University, Harbin, China; Heilongjiang Provincial Key Laboratory of Infection and Immunity, Harbin, China; Key La-boratory of Pathogen Biology, Harbin 150081, P. R. China;

[2] Department of Biomedical Engineering, College of Automation Engineering, Nanjing University of Aeronautics and Astronautics, Nanjing 211106, P.R. China;

Contents

Supplementary Figure S1. Summary of keywords and literatures that were used in MycoResistance database.

Supplementary Figure S2. The architecture and interface of MycoResistance database.

Supplementary Figure S1. Summary of keywords and literatures that were used in MycoResistance database.

		Keyword 2				
		Drug Resistance	Drug Susceptibility	Drug Sensitivity	Drug Response	
Keyword 1	Gene	2,466	604	2,466	604	3,705
	Mutation	2,080	176	1,113	176	
	Genome Variation	113	23	39	12	
	Coding Region	60	22	26	14	
	Non-coding Region	4	1	1	0	
	Intergenic Region	62	25	43	7	
	Non-coding RNA	20	4	12	17	
	Small RNA	31	7	18	7	
		Mycobacteria				
		Keyword 3				

Supplementary Figure S2. The architecture and interface of MycoResistance database. (A) The architecture of MycoResistance. B) The interface of MycoResistance, including 'Search', 'Browse' and 'Download' pages.

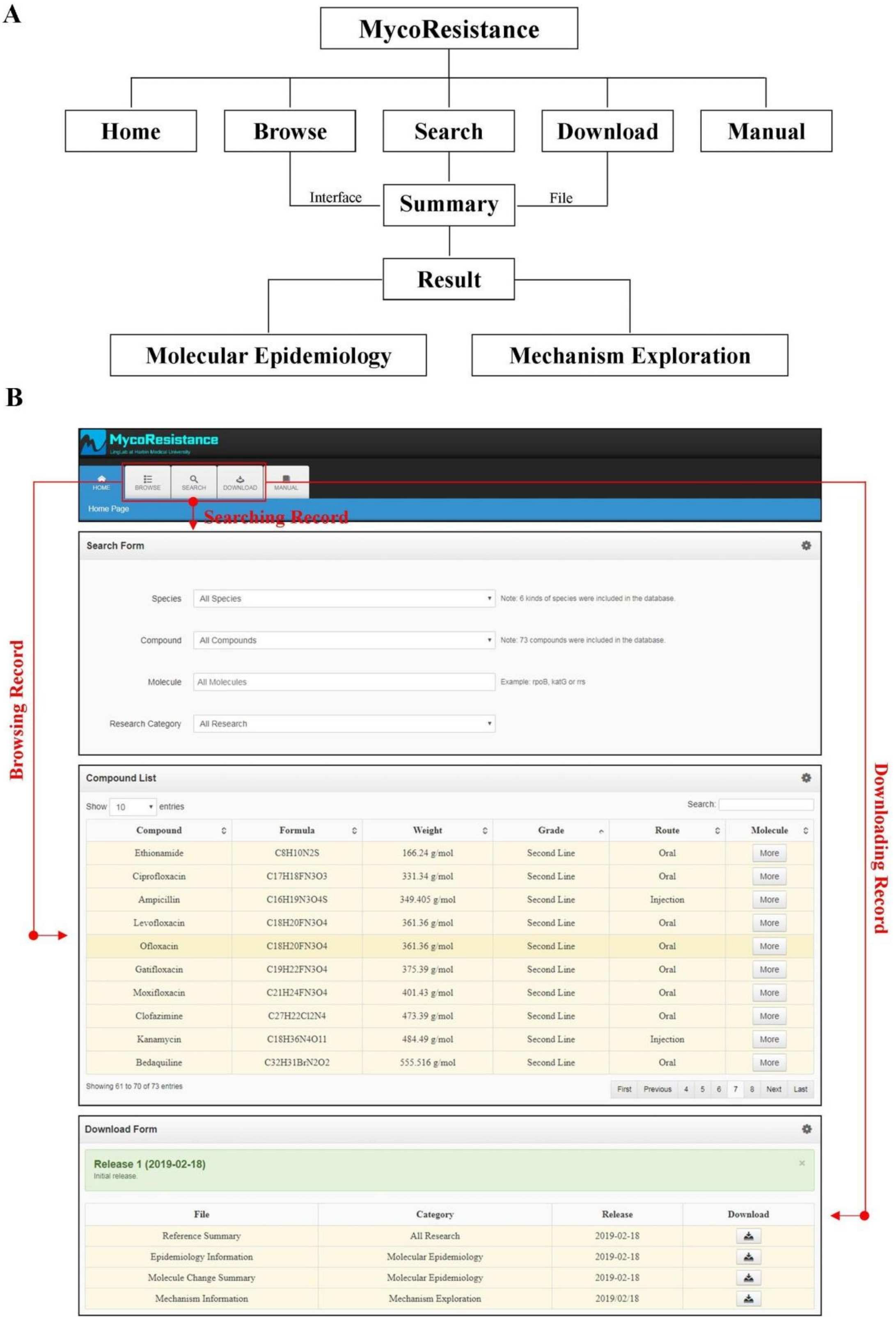

Supplement: Supplementary_File_baz074 [file supplementary_file_baz074.pdf]
